# Increased inflammatory lipid metabolism and anaplerotic mitochondrial activation follow acquired resistance to vemurafenib in *BRAF*-mutant melanoma cells

**DOI:** 10.1038/s41416-019-0628-x

**Published:** 2019-12-10

**Authors:** Teresa Delgado-Goñi, Teresa Casals Galobart, Slawomir Wantuch, Deimante Normantaite, Martin O. Leach, Steven R. Whittaker, Mounia Beloueche-Babari

**Affiliations:** 1 Cancer Research UK Cancer Imaging Centre, Division of Radiotherapy and Imaging, The Institute of Cancer Research, London and The Royal Marsden NHS Foundation Trust, London, SM2 5PT UK; 2 Division of Cancer Therapeutics, The Institute of Cancer Research, London, SW7 3RP UK; 3 Present Address: Department of Psychiatry, University of Oxford, Oxford, OX3 7JX UK

**Keywords:** Melanoma, Nutrient signalling, Metabolomics

## Abstract

**Background:**

BRAF inhibitors, such as vemurafenib, have shown efficacy in *BRAF*-mutant melanoma treatment but acquired-resistance invariably develops. Unveiling the potential vulnerabilities associated with vemurafenib resistance could provide rational strategies for combinatorial treatment.

**Methods:**

This work investigates the metabolic characteristics and vulnerabilities of acquired resistance to vemurafenib in three generated *BRAF*-mutant human melanoma cell clones, analysing metabolic profiles, gene and protein expression in baseline and nutrient withdrawal conditions. Preclinical findings are correlated with gene expression analysis from publicly available clinical datasets.

**Results:**

Two vemurafenib-resistant clones showed dependency on lipid metabolism and increased prostaglandin E2 synthesis and were more responsive to vemurafenib under EGFR inhibition, potentially implicating inflammatory lipid and EGFR signalling in ERK reactivation and vemurafenib resistance. The third resistant clone showed higher pyruvate-carboxylase (PC) activity indicating increased anaplerotic mitochondrial metabolism, concomitant with reduced GLUT-1, increased PC protein expression and survival advantage under nutrient-depleted conditions. Prostaglandin synthase (*PTGES*) expression was inversely correlated with melanoma patient survival. Increases in *PC* and *PTGES* gene expression were observed in some patients following progression on BRAF inhibitors.

**Conclusions:**

Altogether, our data highlight heterogeneity in metabolic adaptations during acquired resistance to vemurafenib in *BRAF*-mutant melanoma, potentially uncovering key clinically-relevant mechanisms for combinatorial therapeutic targeting.

## Background

The mitogen-activated protein kinase (MAPK) pathway, also known as the RAS/RAF/MEK/ERK pathway is involved in the control of cell growth, differentiation and survival in normal physiological conditions.^1,2^ The activity of the proteins involved in this signalling pathway is regulated by a phosphorylation cascade initiated by the activation of tyrosine kinase membrane receptors such as the epidermal growth factor receptor (EGFR). Abnormalities in this kinase cascade play a critical role in the development and progression of cancer, promoting uncontrolled cell replication, evasion of apoptosis, and the ability to invade and metastasise.^2,3^

MAPK pathway activation, which can be driven by mutant BRAF, EGFR, KIT, RAS, MEK or ERK,^4^ is a central step in melanoma pathogenesis. The discovery and characterisation of the molecular underpinnings of melanoma have exposed novel potential therapeutic targets, BRAF being one of the most relevant. Approximately 50% of melanomas harbour activating *BRAF* mutations and over 90% of these give rise to the mutant protein BRAFV600E. This particular mutation has been implicated in different mechanisms of melanoma progression, for example activation of the downstream MEK/ERK pathway, evasion of senescence and apoptosis, induction of angiogenesis, tissue invasion and metastasis and evasion of the immune surveillance.^5^ Accordingly, BRAFV600E was identified as a key target for the treatment of V600E-driven melanoma.^6^

Vemurafenib (PLX4032) was the first drug approved for the treatment of BRAFV600E mutant melanoma, showing improved response rates and both progression-free and overall survival in the clinic.^7^ Unfortunately, the clinical benefits of vemurafenib are short-lived and the majority of patients relapse within 6–7 months.^8^ Molecular mechanisms of resistance to MAPK pathway inhibition can be MAPK-dependent (amplification of *BRAF*, splicing of *BRAF*, *NRAS* mutation, MEK (*MAP2K2*) mutation and loss of function mutations in *NF1*) but may also involve the activation of alternative survival pathways (such as PI3K-Akt-mTOR) through increased tyrosine kinase receptor (RTK)-mediated signalling.^9,10^ Some of the RTKs involved in molecular resistance to BRAF inhibition are the platelet-derived growth factor-receptor-β (PDGFRβ) and the insulin-like growth factor-receptor-1 (IGF-1R).^11^ It is now well established that melanoma cells evoke multiple mechanisms to reactivate the MAPK pathway and that even small changes in the MAPK signalling inputs can overcome the effects of BRAF inhibition. With this in mind, new strategies have been sought to suppress the recovery of MAPK signalling, with the goal of improving overall survival. For example, ERK inhibitors are active against cancers harbouring diverse *BRAF*-mutations, as well as melanomas with BRAF–MEK inhibitor resistance.^2^

Another key player in resistance onset after BRAF inhibition is metabolic reprogramming, one of the hallmarks of cancer.^12,13^ Cancer cells exhibit altered metabolism relative to normal tissues, characterised by an increased dependency on aerobic glycolysis, fatty acid and nucleotide synthesis, and glutaminolysis.^14^ Moreover, lipidomic profiling has revealed unsuspected and recurrent lipid changes at the class and molecular species levels in these cells.^15^ This “metabolic transformation” is considered an enabling hallmark for cancer maintenance and progression that is tightly linked to oncogenic signalling, and as such is being pursued as a promising therapeutic strategy.^16^ In the particular case of melanoma, glycolysis and mitochondrial oxidative phosphorylation (OXPHOS) appear to be the main metabolic pathways involved in tumour maintenance, progression, and drug resistance, but several additional metabolic routes are also known to be activated and promote cell survival under different tumour microenvironments.^12,17^

The aim of this work was to unveil the metabolic characteristics of acquired resistance to BRAF inhibitors in *BRAF*-mutant melanoma cells and identify novel pathways that could provide targeted combinatorial strategies with BRAF inhibitors. Accordingly, we generated three different *BRAF*-mutant human melanoma clones with acquired resistance to vemurafenib and investigated differences in their metabolic characteristics and dependencies.

We show that *BRAF*-mutant melanoma cells with acquired resistance to BRAF inhibition have a heterogeneous metabolic profile that is characterised by an overall lower glycolytic, bioenergetic and phospholipid metabolic activity compared to sensitive cells. Two out of the three resistant clones showed dependency on lipid availability for resistance to vemurafenib, which was associated with higher PGE2 synthesis and mPTGES-1 overexpression. The third clone showed upregulated pyruvate-carboxylase (PC) expression (which was also confirmed in the remaining 2 clones), a switch to PC anaplerotic mitochondrial metabolism and increased survival advantage under glucose and glutamine deprivation.

## Methods

### Cell lines and reagents

The A375 human melanoma cell line was acquired from the American Tissue Type Collection: A375 (BRAFV600E). The B4 and D9 A375 derivative clones resistant to vemurafenib were generated after continuous exposure of the parental line to the drug (~10 × GI_50_) for 6 months, until they started growing progressively. Then the drug was removed from the culture media. To obtain the R6 clone the parental cell line was cultured for four weeks in 1 µM PLX4720 media and resistant clones were subsequently isolated. GI_50_ values for all the clones were tested monthly to confirm the persistence of acquired resistance to the drug

All the clones, including the parental line, were tested by STR profiling on the 7 September 2016. Vemurafenib and [1,2-^13^C]glucose were purchased from Chemietek (Indianapolis, USA) and Sigma–Aldrich (Gillingham, UK) respectively. Gefitinib was purchased from Chemietek (Indianapolis, US). Ninety-six-well plates were from Triplered (UK).

### Cell culture and treatments

Cells were grown as monolayers and routinely cultured as previously described.^18^ For cell viability assays and GI_50_ calculations, the maximum vemurafenib concentrations used were 4.4 µM for the parental A375 cell line and 70 µM for the resistant clones. The maximum concentration of gefitinib was 25 µM for all the clones. Cells were seeded in 96-well plates for 24 h and then the drug was added in double dilutions for the following 72 h. Cell viability was monitored with the cell titre-blue^®^ assay. For drug combination assays, a constant concentration of 6 µM of gefitinib was added to the plates together with the previously described vemurafenib dilutions.

Cell counts were calculated 72 h after seeding 10^6^ cells of each clone in T80 culture flasks. 24 h before harvesting, either 0.01% DMSO or 2 µM vemurafenib were added to the cultures.

For ^13^C-glucose isotopomer analyses, A375 and R6 cells were incubated in media containing 5 mM [1,2-^13^C]glucose, as this concentration is physiologically relevant and has been previously reported to provide similar results to the 25 mM concentration contained in the standard medium used for the ^1^H NMR experiments.^19^ Either 0.01% DMSO or vemurafenib (2 µM, 5xGI_50_ of the parental clone) were added for 24 h.

For nutrient deprivation experiments, cells were seeded in four different media conditions: 5 mM glucose, 1 mM glucose, 1 mM glucose without glutamine, 1 mM glucose without glutamine and pyruvate (48 h before treatment) and either 0.01% DMSO or 2 µM vemurafenib was added for 24 h in the same media. For lipid-restricted conditions, cells were cultured in 1% FBS media + 9% delipidised serum (PAN Bioteck UK Ltd.) to maintain the level of other components such as growth factors and hormones.

### NMR metabolic analyses of cells

Control A375, R6, B4 and D9 cells were extracted with a methanol-chloroform-water method as previously described.^20^ The aqueous fraction was reconstituted in D_2_O using 3-(trimethylsilyl) propionic-2,2,3,3-d4 acid and methylenediphosphonic acid as ^1^H and ^31^P NMR standards, respectively. NMR data were acquired on a Bruker Avance III 500 MHz NMR spectrometer (Bruker Biospin, Ettlingen, Germany). Spectra were processed using MestRe-C version 4.9.9.6 (University of Santiago de Compostela, Spain) and metabolite content was measured by peak integration relative to internal standards and corrected for cell number per sample. Further details on acquisition parameters are provided in the Supplementary material.

### Multivariate analysis of NMR spectroscopy data

^1^H NMR data from the four clones were subjected to unbiased metabolic profiling using partial least squares discriminant analysis (PLS-DA), a method performed after principal component analysis (PCA) to sharpen the separation between groups of observations, determining the variables carrying the class separation information. For this, spectra were processed and data analysed in SIMCA v13.0 (Umetrics-Umeå, Sweden) using a PLS-DA model as previously described.^19^

### Western blotting

Baseline protein levels, target protein expression and phosphorylation levels following BRAF inhibition were assessed by Western blotting using standard conditions as previously described.^20^ Antibody information is provided in the Supplementary section.

### Quantitative real-time PCR (qRT-PCR)

Total RNA was extracted using the RNAeasy kit (Qiagen; Crawley, West Sussex, UK) and 1 µg was reverse transcribed using the High Capacity cDNA Reverse Transcription Kit (Applied Biosystems; Carlsbad, California, USA). Samples were diluted 1:10 and 1 μl of the dilution was used in the Taqman assay, using Taqman universal master mix. Primer information is provided in the Supplementary section.

### Measurement of PGE2 production

The four cell clones were cultured for 48 h, media in the flasks were replenished and samples collected after 24 h. A375 cells were also exposed to 0.01% DMSO or 2 µM vemurafenib 24 h before media collection. PGE2 concentrations in extracellular media were measured using a commercial ELISA kit (Enzo; Exeter, UK) following manufacturer instructions and data corrected for cell number in each culture flask.

### Clinical data

Gene expression and patient survival data for the ‘Skin Cutaneous Melanoma’ TCGA provisional study were obtained from cBioPortal.^21,22^ cBioPortal was used to query the effect of amplification or mRNA overexpression of specific genes on overall survival of 469 melanoma patients by Logrank test.

Gene expression data for pre-treatment and post-progression tumour biopsies from melanoma patients treated with vemurafenib or dabrafenib was from a prior study by Rizos et al.^23^ and downloaded from the Gene Expression Omnibus database (GSE50509).

### Statistical analysis

For metabolite analysis, Student’s *t*-test with Sidak-Bonferroni correction for multiple comparisons (*P* ≤ 0.05) was applied. GI_50_ values, mRNA levels, cell number and PGE2 concentrations were analysed using a single comparison two-tailed unpaired Student’s *t*-test with *P* ≤ 0.05 considered significant. Results are expressed as mean ± SE.

## Results

### *BRAF*-mutant melanoma cell clones with acquired resistance to vemurafenib show heterogeneous metabolic profiles

Three independent clones resistant to vemurafenib were generated from the parental melanoma cell line A375 as described in the Methods section. The GI_50_ values of vemurafenib-resistant clones R6, B4 and D9 (3.9 ± 0.8 µM, 3.2 ± 0.8 µM and 2.5 ± 0.4 µM, respectively) were significantly higher than observed in the sensitive A375 cells (0.2 ± 0.04 µM, *p* < 0.05, Fig. S1A)). In addition, the three vemurafenib-resistant clones showed higher basal levels of ERK phosphorylation, indicating increased MAPK pathway activity (Fig. S1B).

**Fig. 1:**
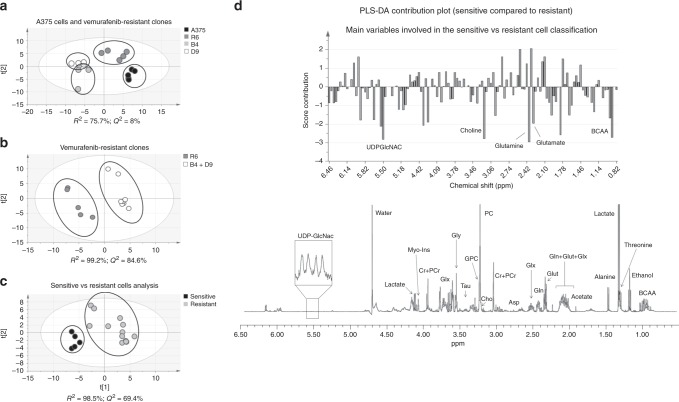
Metabolomic profiling identifies decreased metabolic activity in BRAF-inhibitor-resistant clones. **a** A bi-dimensional PLS-DA score scatter plot showing separate clustering for the sensitive and the resistant clones. **b** Heterogeneity between the three vemurafenib-resistant clones as represented in the score scatter plot. **c** The separation between all the resistant cells, considered as one group, and the sensitive cells. **d** The panel represents a score contribution plot with corresponding changes in the ^1^H NMR peaks (and related metabolites shown in the representative spectrum below) accounting for the differences between sensitive and resistant clones (plot obtained using the Group to Group comparison option in SIMCA). Positive scores represent increased levels while negative scores indicate decreased levels in sensitive relative to resistant cells. Those variables considered as the most relevant for group separation had a contribution coefficient ≥ |2|.

Analysis of the aqueous cell metabolites obtained using ^1^H NMR and a multivariate PLS-DA approach showed clear differences between sensitive and resistant cells on the one hand, and between the three resistant clones themselves, on the other hand, consistent with heterogeneity (Fig. 1a). Resistant clones B4 and D9 were clustered close to each other and separated from clone R6 suggesting two different metabolic groups among the resistant cells. This was confirmed with a PLS-DA analysis comparing R6 cells with B4-D9 clones pooled together, indicating a very clear separation between the two groups (Fig. 1b) mainly based in branched-chain amino acids (BCAA, 0.9 ppm), glutamate (2.3 ppm), glutathione (2.54 ppm) and phosphocholine (3.22 ppm) content (Fig. S2). To characterise the main metabolic differences between resistant and sensitive cells, the data from the three resistant clones were pooled and compared to the parental cells (Fig. 1c) obtaining a good classification model based on four principal components where the first three explained 42.4% of the variability in the data (PC1: 17.9%, PC2: 12.2%, PC3: 13.3%). The main metabolic differences between sensitive and resistant cells (summarised in Fig. 1d) were based on their glutamine (2.38 ppm), glutamate, choline (3.18 ppm), BCAA and uridine diphosphate N-acetylglucosamine (UDPGlcNAC, 5.5 ppm) content.

A univariate analysis of the ^1^H NMR spectra from the four clones was performed as previously described^19,24^ to validate the multivariate analysis results. This confirmed that the resistant cells produce significantly less TCA metabolism-related metabolites (acetate, fumarate), pentose phosphate pathway (PPP) (ribose), glutamine (glutamine, glutamate, glutathione), hexosamine (UDP-GlcNAC) with a trend towards decreased phosphocholine (*p* = 0.07) (Table S1). ^31^P NMR analysis confirmed a significant decrease in choline containing metabolites and NTPs in the resistant clones, correlating with a lower metabolic and bioenergetics state. Cell counts obtained 72 h post-seeding in all the cell lines showed a lower proliferation rate in the resistant cells (50.7 ± 7.8%, 62.2 ± 8.2% and 49.7 ± 8.5% with respect to the parental cell line (*p* ≤ 0.05)), consistent with the lower bioenergetics profile (Fig. S1C, Table S1). Significant differences between R6 and B4-D9 clones were only confirmed in the ^31^P NMR univariate approach (Table S2) suggesting an even lower bioenergetic profile in B4 and D9 clones.

Altogether these data reveal metabolic heterogeneity between vemurafenib-resistant clones derived from A375 cells with a common shift towards an overall decrease in cellular metabolic activity, lower utilisation of glucose, glutamine and pyruvate, and a decrease in bioenergetics and phospholipid-related metabolites when compared to parental cells.

### Lipid metabolism as a potential Achilles Heel for acquired resistance in *BRAF*-mutant melanoma

**Fig. 2:**
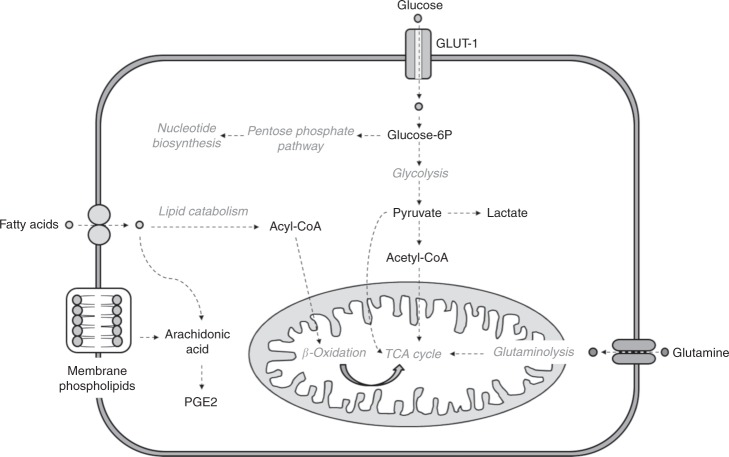
Schematic representation of the main metabolic routes investigated by nutrient withdrawal in sensitive and resistant cells. Glucose, glutamine, pyruvate and lipids were depleted from the media and the metabolic and molecular responses induced in the cells were characterised. GLUT-1, glucose transporter; PGE2, prostaglandin E2.

**Fig. 3:**
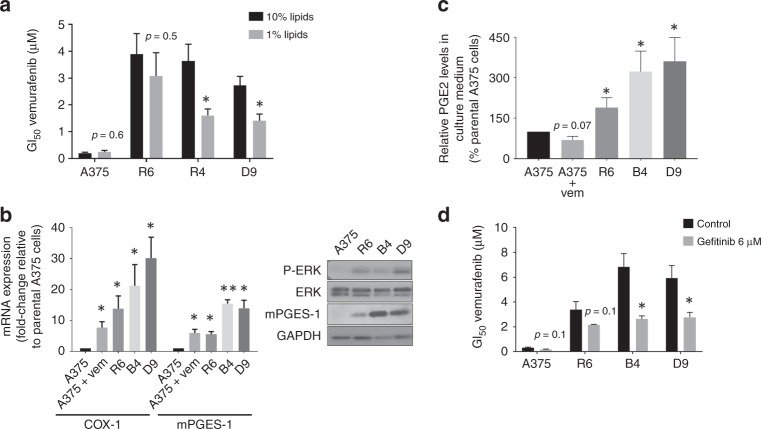
Resistance to BRAF inhibition is associated with a greater dependency on lipids and EGFR signalling. **a** GI_50_ values for vemurafenib in the 4 clones growing under 10 and 1% lipid media. B4 and D9 clones become more sensitive to the drug under lipid-depletion. **b** On the left, mRNA expression of COX-1 and mPTGES-1 in the three resistant clones and treated (2 µM vemurafenib) sensitive cells with respect to the parental clone A375. On the right, protein expression levels of PGE2 and MAPK related proteins in a representative replicate of the four clones under standard media conditions. **c** Extracellular PGE2 excretion after 24 h of fresh media addition in the four clones (% with respect to A375-sensitive cells). **d** GI_50_ values for vemurafenib in the four clones when combined with 6 µM gefitinib. *p < 0.05, **p < 0.01.

Taking into account the common metabolic shift towards lower metabolic activity and bioenergetics in resistant cells described in the previous section, the parental cells and the three resistant clones were subjected to different metabolic challenges involving the main metabolic pathways and energy substrates in cancer cells (Fig. 2).^25^ Lipids are a significant source of signalling messengers as well as bioenergy in cancer cells.^15^ We thus assessed the dependency of response to vemurafenib in sensitive and resistant cells on lipid availability by growing cells under conditions of reduced exogenous lipid content. Two resistant clones (B4 and D9) showed a two-fold decrease in vemurafenib GI_50_ values under lipid depletion (*p* = 0.02, Fig. 3a). The resistant clone R6 and the sensitive parental A375 cells showed no significant shift in vemurafenib GI_50_ under the same conditions.

Assessment of protein expression related to lipid oxidation (ACAD9) and biosynthesis (acetyl-CoA carboxylase, ACC) showed no differences between vemurafenib-resistant and sensitive cells (Supplementary Material Fig. S3). This finding indicated that the lipid dependency in the resistant cells could be linked to a cellular process different from energy release through lipid catabolism or fatty acid biosynthesis. As described in the previous section, ERK phosphorylation was upregulated in the three vemurafenib-resistant clones relative to the sensitive cells (Fig. S1B), indicating reactivated MAPK signalling in vemurafenib-resistant cells. It is well known that lipids (e.g. arachidonic acid (AA)) are important substrates for the COX-PGE synthase pathway, and it has been reported that a crosstalk between COX-derived PGE2 and EGFR can induce ERK activation.^26^ Moreover, extensive evidence implicates both COX and EGFR signalling in carcinogenesis.^27,28^ Thus, we investigated whether the lipid dependency observed in two of the three resistant clones was concomitant with increased PGE2 synthesis. Analysis by qRT-PCR of COX-1, COX-2 and PGE synthase (mPTGES-1) mRNA expression showed that the three resistant clones had significantly increased expression of mPTGES-1, COX-1 but not COX-2 in comparison with the parental cells (Fig. 3b). Western blotting confirmed higher expression of mPTGES-1 at the protein level in the B4 and D9 resistant clones and to a lesser extent in clone R6 (Fig. 3b) although no changes in COX-1 protein expression were observed (data not shown). Interestingly, the two resistant clones that were re-sensitised to vemurafenib following lipid depletion (B4 and D9) were those with the highest mPTGES-1 protein expression. Moreover, under BRAF inhibition the sensitive cell line A375 showed upregulated mRNA expression of both COX-1 and mPTGES-1 (Fig. 3b) suggesting that this could be a drug-induced effect that becomes a permanent feature in cells with acquired resistance.

To confirm functionality of the induction in mPTGES-1, we assessed the release of PGE2 to the extracellular media after 24 h of cell incubation in fresh media. Our data indicated that all three resistant clones produced significantly more PGE2 than the sensitive cells, with B4 and D9 showing the largest increases, in line with the largest induction in mPTGES-1 (Fig. 3c).

In order to assess the possibility of EGFR-MAPK signalling pathway involvement in vemurafenib resistance in our clones, we tested combinatorial treatment with the EGFR inhibitor gefitinib and vemurafenib in the four cell lines. Gefitinib re-sensitised B4 and D9 cells to vemurafenib decreasing significantly their GI_50_ value for the BRAF inhibitor from 6.8 ± 1.1 µM to 2.6 ± 0.3 µM and from 5.9 ± 1.0 µM to 2.8 ± 0.4 µM, respectively, (*p* ≤ 0.05, Fig. 3d). There was a trend towards decreased vemurafenib GI_50_ values with gefitinib treatment in R6-resistant cells, but this did not reach statistical significance (*p* = 0.1, Fig. 3d).

In summary, these data show that the B4 and D9, but not R6, clones are re-sensitised to vemurafenib under reduced exogenous lipid content concomitant with upregulated mPTGES-1 expression and PGE2 production. Combination treatment with the EGFR inhibitor gefitinib re-sensitised both these clones to BRAF inhibition with vemurafenib, suggesting a potential role for EGFR signalling in acquired resistance.

### Vemurafenib-resistant cells increase anaplerotic mitochondrial activation whilst downregulating glycolytic metabolism

According to the previous results described, the common metabolic signature of acquired resistance to vemurafenib characterised in the three resistant clones could not be related to the same molecular and metabolic alterations. The differential response to lipid withdrawal between the B4-D9 and the R6 clones suggested the involvement of heterogenous mechanisms of resistance converging in some common metabolic features. Thus, we decided to further investigate the metabolic processes underpinning drug resistance in R6 cells exploring the role of glucose as another key metabolic substrate for cancer cells. We have previously reported that *BRAF*-mutant cells upregulate anaplerotic mitochondrial metabolism through PC activation under vemurafenib treatment, and that this metabolic rewiring confers a survival advantage under nutrient-depleted conditions.^19^ Thus, we focused our experiments on the characterisation of glucose metabolic flux in sensitive and R6 cells and its implications in cell survival under different metabolic challenges using ^13^C-glucose labelling and isotopomer analysis. For this, parental A375 and the R6-resistant clone were incubated in [1,2-^13^C]glucose.

**Fig. 4:**
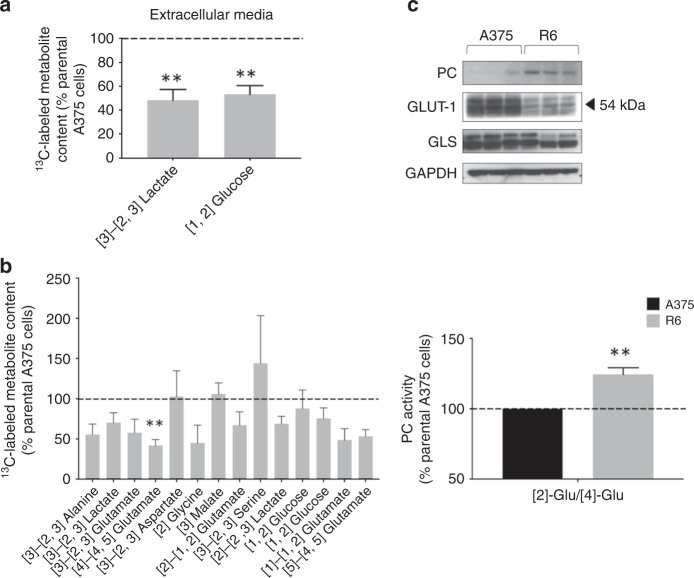
Vemurafenib-resistant cell clones exhibit decreased glycolytic metabolism and enhanced mitochondrial anaplerotic metabolism. **a** Resistant R6 cells produce significantly less lactate^E^ and show a significantly lower glucose uptake in comparison to A375 cells. **b** R6 cells produce significantly less glutamate ([4]-[4,5] glutamate) than sensitive cells but maintain similar fumarate and acetate levels despite a lower glucose consumption rate, indicating a potential upregulation in TCA activity non-glutamine or PDH dependent. Anaplerotic TCA activity is significantly higher in R6 cells compared to the parental clone (*p* = 0.01). **c** The molecular analysis of three biological replicates from A375 and R6 cells revealed a lower GLUT-1 and GLS (KGA and GAC isoforms) expression and a higher PC expression in vemurafenib-resistant cells in comparison to sensitive cells. **p < 0.01.

Media metabolite content analysis prior to and 24 h following [1,2-^13^C]glucose administration showed that R6 cells had significantly lower glucose uptake (53.2 ± 7.3%, *p* = 0.01) paralleled with lower lactate excretion to the media (48.3 ± 8.9%, *p* = 0.01) when compared to drug sensitive A375 cells (Fig. 4a). Cellular extracts metabolite analysis revealed significantly lower levels of [4]-[4,5-^13^C]glutamate in resistant cells (41.8 ± 7.5%, *p* = 0.02) as well as a tendency to a fall in lactate production, although this was not statistically significant (70.3 ± 12.2%, *p* = 0.08; Fig. 4b). These results were consistent with the ^1^H NMR analysis, suggesting lower glycolytic activity in the R6 cells compared to the parental clone. However, [3]-[2,3-^13^C]aspartate and [3-^13^C]malate levels were not significantly different from those detected in sensitive cells (102.8 ± 31.9%, *p* = 0.9 and 105.8 ± 13.9%, *p* = 0.7, respectively) despite the significant reduction in glucose uptake, indicating a relative increase in the TCA activity of resistant cells. This could be explained by a significant increase in the ratio of anaplerotic/oxidative mitochondrial activity in R6 cells, as shown by the ratio [2-^13^C]Glutamate/[4-^13^C]Glutamate that reports the activity of PC versus PDH (124.4 ± 4.7%, *p* = 0.01; Fig. 4b).^29^ No significant differences were found in metabolites related to the PPP activity between sensitive and vemurafenib-resistant cells. Other metabolites such as alanine or glycine showed a trend towards a decrease in R6 cells but data dispersion within groups, probably due to the lower SNR related to the NMR signal of those metabolites, was too high to reach statistical significance (Fig. S4).

Analysis of the protein expression of metabolic enzymes in A375 and R6 cells corroborated the findings described above (Fig. 4c); relative to parental cells, vemurafenib-resistant cells showed higher PC protein expression at baseline conditions (also observed in B4 and D9 cells, Fig. S5), concomitant with enhanced anaplerotic mitochondrial metabolism. R6-resistant cells also expressed reduced levels of GLUT-1, in line with reduced glucose transport, and glutaminase, consistent with reduced glutamine metabolism, also shown by the ^1^H NMR profiling.

Overall, these data show that R6 vemurafenib-resistant cells exhibit decreased glycolytic metabolism and enhanced mitochondrial anaplerotic metabolism through PC activation that is associated with increased PC protein expression.

### Vemurafenib-acquired resistance in R6 cells decreases dependency on glucose, glutamine and pyruvate

The significance of the metabolic changes associated with vemurafenib resistance in R6 cells was next investigated by exposing the sensitive and R6 clones to different metabolic challenges, depleting key nutrients in the culture media: glucose, glutamine and pyruvate (as depicted in Fig. 2). The purpose was to assess whether the altered metabolic phenotype would confer a survival advantage to the resistant cells and if the withdrawal of any of the selected metabolites could affect their viability.

**Fig. 5:**
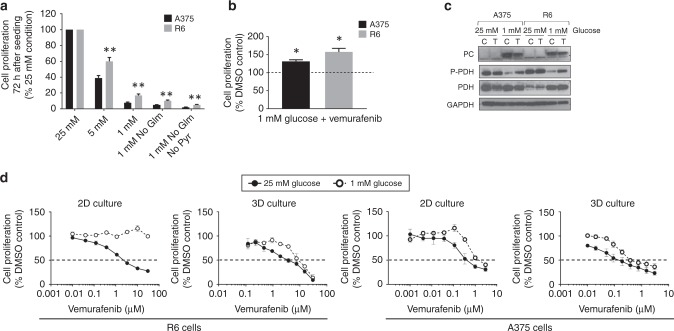
A decreased dependency on glucose, glutamine and pyruvate is observed in vemurafenib-resistant cells. **a** Cell numbers after 72 h of seeding A375 and R6 cells in different nutrient conditions. **b** Cell numbers in both A375 and R6 clones after 72 h growing in 1 mM glucose media with 2 µM vemurafenib for the last 24 h, compared to DMSO-treated cells growing in the same glucose conditions. **c** TCA-related protein expression in control and treated samples (2 µM vemurafenib) from both clones in 25 or 1 mM conditions. **d** GI_50_ curves for R6 2D and 3D cell cultures with vemurafenib in 25 mM or 1 mM glucose media. *p < 0.05, **p < 0.01.

Cell numbers were recorded after 72 h of glucose depletion (from 5 to 1 mM), glutamine or pyruvate withdrawal (Fig. 5a). R6 cells showed a significantly lower growth rate than parental A375 cells (79.4 ± 2.7, *p* = 0.02) in standard medium conditions (25 mM glucose), in line with the earlier findings (Fig. S1C). However, R6 cells showed an increased proliferation rate compared to the parental clone under all the nutrient-depleted conditions indicating decreased dependency on glucose, glutamine and pyruvate, consistent with the lower utilisation of these metabolites as detected by ^1^H NMR (Table S1 and Fig. 1). Interestingly, both sensitive and R6 cells growing in 1 mM glucose media under BRAF inhibition (vemurafenib 2 µM) showed increased proliferation relative to DMSO-treated cells (Fig. 5b, 131.9 ± 4.1% and 157.5 ± 10.1%, respectively; *p* < 0.05). This finding is in agreement with our previous results showing that BRAF inhibition improves *BRAF*-mutant melanoma survival in nutrient-depleted conditions.^19^

The molecular profile of A375 and R6 cells revealed that in standard 25 mM glucose conditions resistant cells express higher levels of PC compared to sensitive cells (Fig. 5c), in agreement with the data presented in Fig. 4c. Under glucose deprivation (1 mM) both clones showed increased expression of PC and dephosphorylated (activated) PDH, indicating that the main metabolic routes to produce energy in this setting involved mitochondrial metabolism. Under BRAF inhibition the expression of PC was maintained in both clones whereas PDH showed inactivation through phosphorylation, indicating that vemurafenib induces mitochondrial anaplerotic metabolism, as previously reported.^19^

To assess the implication of mitochondrial activation induced under glucose deprivation on cell response to BRAF inhibition, growth inhibition assays were carried out with vemurafenib and A375 and R6 cells in either 25 mM or 1 mM glucose media. These measurements were also performed in cells grown as 3D spheroids (i.e. in nutrient availability gradients) in order to better mimic the metabolic conditions in the tumour microenvironment and their influence on mitochondrial activation in vivo. The data showed that R6 cells were significantly more resistant to vemurafenib in low glucose media particularly when grown in 2D, with GI_50_ values increasing from 2.6 ± 0.3 µM to > 30 µM (*p* = 0.0001) in 2D and from 4.2 ± 1.4 µM to 9.7 ± 1.7 µM (*p* = 0.07) in 3D cultures (Fig. 5d). A similar, albeit less profound trend, was observed in the sensitive A375 cells (GI_50_ values increased from 0.6 ± 0.2 µM to 1.3 ± 0.8 µM (*p* = 0.5) in 2D and from 0.2 ± 0.1 µM to 0.9 ± 0.6 µM (*p* = 0.3) in 3D (Fig. 5d)). Thus, induction of mitochondrial metabolism, particularly via PC, may be involved in conferring resistance to vemurafenib.

In summary, vemurafenib-resistant cells show upregulation of mitochondrial PC metabolism which, In R6 cells at least, confers a survival advantage in nutrient-restricted conditions, further reducing cell sensitivity to vemurafenib.

### Correlation of altered metabolism with patient survival and BRAF-inhibitor treatment outcome in clinical melanoma

**Fig. 6:**
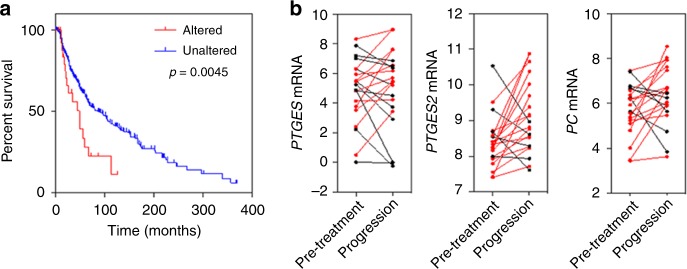
Elevated expression of *PTGES*/*PTGES2* is associated with poor survival of melanoma patients and acquired resistance to BRAF inhibition. **a** Overall survival in 469 patients affected by melanoma tumours with or without genetic alterations (amplification or mRNA overexpression) in the *PTGES* or *PTGES2* genes. Alterations in *PTGES* or *PTGES2* (red line, z-score > 2) correlated with a significantly lower survival (*p* = 0.0045). **b** Expression of *PTGES*, *PTGES2* and *PC* mRNA in pre-treatment and post-progression tumour biopsies from melanoma patients treated with vemurafenib or dabrafenib (red lines and symbols indicate increased expression in the post-progression biopsy relative to the pre-treatment biopsy).

The clinical relevance of the metabolic signatures of vemurafenib-resistant cells identified here was next examined using data from melanoma patients reported in the CBioPortal database. The presence of *PTGES*, *PTGES2*, *PTGES3* and *PC* gene amplification or elevated expression (z-score > 2) was analysed in relation to survival in a group of 469 patients. Interestingly, 5.5% of patients had tumours with amplification of *PTGES* or *PTGES2* or increased expression of the mRNAs they encode. In these subjects, overall survival was significantly decreased with median survival of 85 months in unaffected patients and of 49 months in affected patients (Fig. 6a), suggesting the potential clinical relevance of our findings and indicating that PGE2 synthesis could be a promising target for combinatorial therapy. No clear correlation was found between *PC* or *PTGES3* expression and survival in this dataset. Furthermore, gene expression analysis of pre-treatment and post-progression biopsies from a published cohort of melanoma patients treated with the BRAF inhibitors vemurafenib or dabrafenib indicated that the mRNA expression of *PTGES* or *PTGES2* as well as *PC* was increased in the tumours of some patients who experienced progressive disease (Fig. 6b).^23^ Therefore, it is conceivable that elevated *PTGES/PTGES2* and/or *PC* expression may contribute to BRAF-inhibitor resistance in melanoma patients.

## Discussion

Acquired resistance to BRAF-MEK-ERK signalling inhibitors, which occurs through ERK signalling-dependent and -independent mechanisms, has been a major challenge for the treatment of *BRAF*-mutant melanomas.^11,30,31^

Previous findings related to metabolic mechanisms of acquired resistance in melanoma report an increased dependency on mitochondrial metabolism either through PC^19^ or glutamine metabolism,^32^ and increased glycolytic metabolism.^33^ The disparity in the findings reported highlights heterogeneity and the need to investigate several resistant clones to gain a wider understanding of acquired-resistance mechanisms. The aim of this work was to evaluate the metabolic characteristics and dependencies of different clones of *BRAF*-mutant cells following acquisition of resistance to the BRAF-inhibitor vemurafenib and assess their clinical relevance.

The three resistant clones investigated showed inter-clone metabolic heterogeneity characterised by differences in BCAA, glutamate, glutathione and phosphocholine content but overall the vemurafenib-resistant clones exhibited a lower metabolic activity profile compared to the parental cells as indicated by metabolite levels related to TCA (acetate, fumarate), pentose phosphate pathway (PPP) (ribose), glutamine metabolism (glutamine, glutamate, glutathione) and hexosamine metabolism (UDP-GlcNAC). This was concomitant with lower proliferation rates, in agreement with previous studies reporting decreased susceptibility to various anti-cancer agents in low-cycling melanoma cells.^34,35^

The common metabolic signature of acquired resistance identified in the three vemurafenib-resistant clones was investigated using different metabolic challenges involving the most relevant substrates for cancer cells’ proliferation and maintenance.^25^ Thus, lipid metabolism was investigated as a relevant energy source that is often upregulated in cancer cells.^15^ Our data show that two out of the three resistant clones displayed re-sensitisation to vemurafenib when cultured in lipid-depleted media, potentially implicating lipid metabolism in vemurafenib resistance in these clones. Further work involving lipid transporters such as CD36 or FATP is required to assess if this dependency in *BRAF*-mutant melanoma is based on lipid uptake or biogenesis processes.^36,37^

In addition to their role as energy fuels, lipids orchestrate signal transduction cascades that regulate pro-oncogenic and apoptotic pathways in different stages of carcinogenesis.^15^ As our resistant clones showed upregulated ERK phosphorylation (i.e. increased baseline ERK signalling) in comparison to the parental cells, we investigated the hypothesis pointing at potential links between MAPK signalling and lipid metabolism that could lead to the acquired-resistance phenotype. It is well known that membrane phospholipids are the main substrate for the COX-PGE2 signalling pathway^38^ and one of the links between aberrant signalling and metabolic reprogramming in cancer cells is the crosstalk between EGFR activation and PGE2 synthesis from fatty acids through COX activation (COX-1 and COX-2).^39,40^ Our data show that the resistant clones have upregulated mPTGES-1 expression, leading to increased PGE2 production that was more pronounced in B4 and D9 clones, showing vemurafenib re-sensitisation under lipid depletion, supporting the involvement of this metabolic shift in acquired resistance to vemurafenib in these clones.

Rapid EGFR-mediated reactivation of the MAPK pathway has been connected to the relative insensitivity of *BRAF*-mutant colorectal carcinoma cells (CRCs) to vemurafenib, suggesting that combined BRAF and EGFR inhibition could be a promising therapeutic strategy to overcome one of the mechanisms of resistance to BRAF targeted therapies.^41^ Indeed, we show that co-treatment with an inhibitor of EGFR (as the last effector in the COX-PGE2-EGFR crosstalk) re-sensitises *BRAF*-mutant melanoma cells with acquired resistance to vemurafenib. Whether this response could be affected or modulated by the availability of exogenous lipids is a possibility that requires further investigation, as others have reported both an enhancing or a suppressive role for lipid supplementation in EGFR or BRAF inhibition.^42,43^ An interesting aspect to be pursued in future work involving the full characterisation of lipid metabolism in acquired resistance to BRAF inhibitors would be the link between inflammatory lipid dependency and EGFR-ERK signalling activation. The confirmation of a crosstalk between these pathways could provide a potential target for combinatorial therapeutic strategies for *BRAF*-mutant melanoma either at the level of EGFR and/or PGE2 production.

Activation of PGE2 synthesis in *BRAF*-mutant melanoma has also been shown to modulate the tumour microenvironment, specifically leading to increased immune evasion via recruitment of immune suppressive cells.^40^ Our data suggest that the increased production of PGE2 following acquired resistance to vemurafenib could dampen immune surveillance in the tumour microenvironment further contributing to uncontrolled tumour growth.

Further analysis of the R6 clone, which did not show dependency on lipid metabolism, confirmed lower glycolytic activity in these cells, in agreement with ^1^H NMR metabolic findings. However, levels of TCA metabolites such as aspartate and malate were maintained with respect to sensitive cells despite lower glucose consumption indicating a relative increase in resistant cell mitochondrial metabolism. The mitochondrial activation in resistant cells was confirmed by an increase in the ratio anaplerotic/oxidative mitochondrial activity, estimated from the ratio [2-^13^C]Glutamate/[4-^13^C]Glutamate, suggesting higher PC activity.^29^ These findings are in line with previous studies reporting elevated mitochondrial bioenergy metabolism in slow-cycling melanoma resistant cells characterised by an upregulation of proteins related to cell respiratory electron transport.^35^ Our results also correlate with previous work reporting that *BRAF*-mutant cells show a significant reduction in glycolysis and an upregulation of the mitochondrial anaplerotic metabolism under vemurafenib treatment,^19^ suggesting that this metabolic profile is maintained as a permanent feature in acquired resistance. The upregulation in TCA cycle activity was not observed in the ^1^H NMR data because this analysis reports on the global metabolic content of the cell, which depends on many pathways and the net change between *de novo* synthesis and breakdown/utilisation. In contrast, the dynamic ^13^C NMR flux detects de novo synthesis from ^13^C-glucose, which may not necessarily lead to changes in the total ^1^H NMR-measured metabolite pool.

Molecular analysis of parental and R6 cells revealed lower expression of the glucose transporter GLUT-1 and of glutaminase, a key enzyme in glutamine metabolism, consistent with lower glycolytic and glutamine metabolism in the resistant cells. An increase in PC expression was consistent with a higher anaplerotic TCA activity compared to the parental clone and this was also observed in the other two resistant clones, suggesting that it is a common feature in this model.

The ^13^C isotopomer and molecular analyses indicated that R6 cells are less dependent on glucose and glutamine metabolism than sensitive cells. It has been reported that dependence on glycolysis and a lack of functional mitochondrial respiration increases melanoma sensitivity to BRAF inhibitors^44^ and that an increased dependency on mitochondria for survival is a characteristic of acquired resistance to BRAF inhibitors.^45^ However, in some cases dependence on increased oxidative metabolism of resistant melanoma cells is associated with a switch from glucose to glutamine metabolism.^45^ Here we report a metabolic shift from glycolysis to mitochondrial activation in resistant cells via anaplerotic PC activity. Previous reports have linked increased PC flux in glioblastoma and non-small-cell lung cancer cells to reduced dependency on glutamine,^46,47^ in line with our observations. Indeed, we have previously shown that a shift from glycolysis to anaplerotic mitochondrial metabolism occurs following response to vemurafenib in *BRAF*-mutant melanoma cells, improving survival in nutrient-depleted conditions, potentially facilitating the emergence of drug resistant clones.^19^ Here we show that under low glucose conditions (1 mM) and in the absence of glutamine and pyruvate, resistant cells are able to proliferate better than the sensitive clone. Thus, PC activation should be further investigated as a potential metabolic target to overcome acquired resistance onset in *BRAF*-mutant melanoma. Moreover, future investigations involving those metabolic pathways showing potential differences in R6 cells (alanine and serine-glycine) could unveil new potential metabolic targets in vemurafenib-acquired resistance as previously reported.^48^

Finally, the clinical relevance of the metabolic signatures characterising acquired-resistance to vemurafenib in our clones was examined using gene expression and survival data from publicly available datasets of melanoma patients. Amplification or overexpression of *PTGES*/*PTGES2* in melanoma samples was associated with a significantly lower patient survival, emphasising the significance of our findings. Notably, given our observation that *PTGES*/*PTGES2* mRNA expression (as well as *PC* mRNA in some cases) is increased in post-progression biopsies from melanoma patients treated with vemurafenib or dabrafenib, there is potential for altered metabolic programs, as we have described, to contribute to the acquisition of BRAF-inhibitor resistance in patients. Future work is necessary to assess the generalisability of our findings using resistant clones from different parental cell lines and to address the potential of combining emerging inhibitors of these enzymes with BRAF inhibitors to curb the development of resistance.^49,50^

In conclusion, our work shows that acquired resistance to BRAF inhibitors in *BRAF*-mutant melanoma is characterised by lower glycolytic and bioenergetic metabolism. Importantly, we show heterogeneity in metabolic dependencies of the resistant cells involving increased inflammatory lipid metabolism through PGE2 and/or increased mitochondrial PC activity that are observed clinically, and which could have significant consequences for promoting cancer cell survival in the tumour microenvironment. The significance of these metabolic changes as potential therapeutic targets for combination treatment to improve the response to vemurafenib, and potentially other BRAF-MEK-ERK signalling inhibitors, warrants further investigation.

## Supplementary information

CM-2019-2008R Supplementary material

## Data Availability

The datasets generated and analysed during the current study are not publicly available but available from the corresponding authors on reasonable request.
