# Supplementary material for: Increased inflammatory lipid metabolism and anaplerotic mitochondrial activation follow acquired resistance to vemurafenib in BRAF-mutant melanoma cells
Source: Br J Cancer. 2019 Dec 10;122(1):72–81. doi: 10.1038/s41416-019-0628-x (PMC6964672; doi:10.1038/s41416-019-0628-x)
Supplement: Supplementary file 1 — CM-2019-2008R Supplementary material [file 41416_2019_628_MOESM1_ESM.docx]

**Increased** **inflammatory lipid metabolism and anaplerotic mitochondrial activation follow acquired resistance to vemurafenib in *BRAF*-mutant melanoma cells.**

**SUPPLEMENTARY MATERIAL AND METHODS**

**NMR metabolic analysis of cells**

Cells were extracted with a methanol-chloroform-water method and lyophilized samples of the aqueous fraction reconstituted in 540 µL of D2O containing 5% of 3-(trimethylsilyl) propionic-2,2,3,3-d4 acid (TSP, internal reference and quantification standard for ^1^H NMR). Following ^1^H NMR acquisition, EDTA and methylenediphosphonic acid (MDPA, internal standard) were added to a final concentration of 10 and 0.86 mM, respectively, at pH 8.2 for ^31^P NMR analysis. Media fractions were collected from control and treated cells, centrifuged to discard debris and 1ml of each freeze-dried and re-suspended in D2O-TSP.

**NMR data acquisition and processing**

For ^1^H NMR, a 30° flip angle, a 2s relaxation delay (RD), a spectral width of 13 ppm and 64 k data points were used under conditions of water signal suppression for aqueous samples. ^31^P NMR spectra were acquired using power gated composite pulse ^1^H decoupling, a 30° flip angle, a 1s RD, a spectral width of 100 ppm and 32 k data points. ^13^C NMR data were acquired using power gated composite pulse ^1^H decoupling, 33 k data points, a 250 ppm spectral width, a repetition time of 6 s and a 30° flip angle. A line broadening of 0.2 Hz was applied for ^1^H, 2Hz for ^31^P spectra and 5 Hz for ^13^C spectra.

**Western blotting**

The following primary antibodies were used: anti COX-1, anti COX-2 (R&D Systems; Minneapolis, US ), anti-prostaglandin E2 syntahse (mPGES-1)(Cayman chemical; Michigan, US), anti-ERK1/2 (total and phosphorylated), anti-pyruvate dehydrogenase (PDH, total and phosphorylated) (Cell Signaling Technology; Danvers, MA, USA), anti-GLUT-1, anti-glyceraldehyde-3-phosphate dehydrogenase (GAPDH) (Millipore, Nottingham, UK), anti-glutaminase (GLS)(Proteintech Europe; Manchester, UK) and anti-pyruvate carboxylase (abcam, Cambridge, UK). The secondary antibodies used were anti-rabbit (GE Healthcare Life Sciences; Buckinghamshire, UK), anti-goat (Santa Cruz Biotechnology; Santa Cruz, CA, USA) and anti-mouse Immunoglobulins/HRP (Dako A/S, Glostrup, Denmark).

**Quantitative real-time PCR (qRT-PCR)**

The Hs00377726_m1, Hs00153133_m1 and Hs00610420_m1 assays were used for the COX-1, COX-2 and mPGES-1 genes respectively (gene specific primers and FAM labelled probe) multiplexed with the endogenous control assay for β-ACTIN gene (β-ACTIN-specific primers and VIC/TAMARA-labelled probe; Applied Biosystems). mRNA levels of each gene were determined for each sample in the same well on the ABI 7900HT relative to those of β-ACTIN and data processed by expressing changes as the differences in the threshold cycle (2-∆∆Ct), inversely proportional to the amount of target mRNA per sample.

**SUPPLEMENTARY FIGURES**

**FIGURE S1**

**
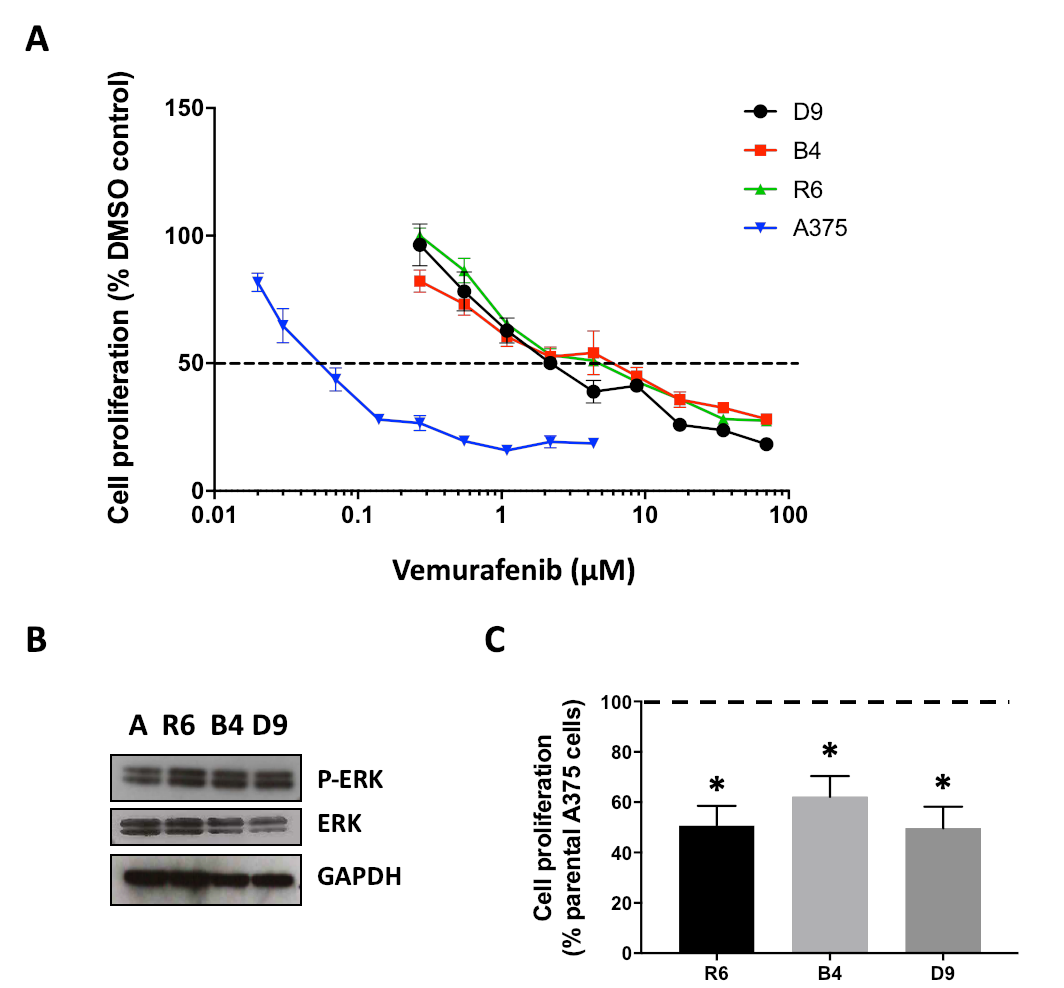
**

**Figure 1**: **Generation and characterization of BRAF inhibitor-resistant A375 cell clones. A**) GI_50_ curves for vemurafenib sensitive (4.4 µM maximum dose) and resistant clones (70 µM maximum dose). **B**) Baseline P-ERK and ERK expression levels in sensitive and resistant cell lines. **C**) Changes in cell counts of the 3 vemurafenib-resistant clones compared to A375 cells after 72 h of seeding.

**FIGURE S2**

**
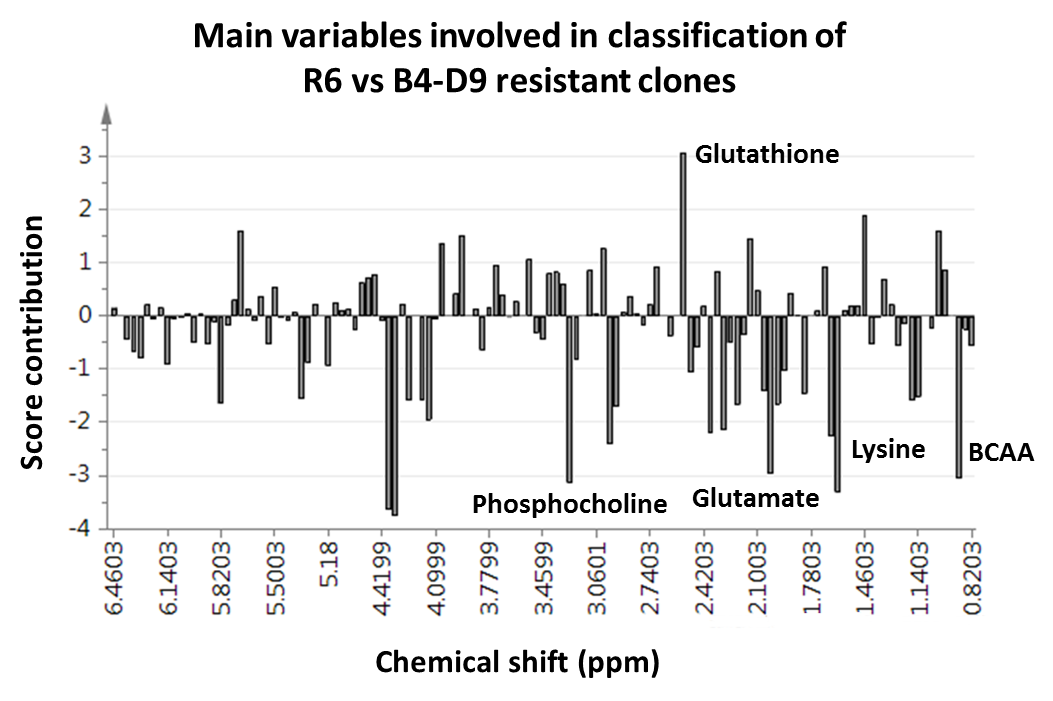
**

**Figure S2: Multivariate analysis of NMR metabolic profiles from the three vemurafenib-resistant clones.** Score contribution plot representing the main metabolic differences allowing the clustering or the R6 clone versus B4-D9 clones, as displayed in Figure 1B in the main manuscript.

**FIGURE S3**

**
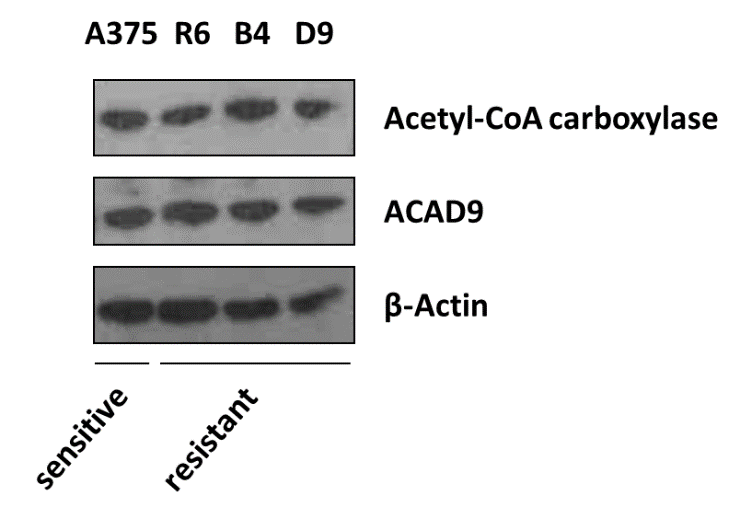
**

**Figure S3: Lipid anabolism and catabolism assessment in sensitive and resistant clones.** Protein expression of enzymes related to fatty acid synthesis (Acetyl-CoA carboxylase (ACC)) and lipid β-oxidation (ACAD9) in A375 (A) cells and three vemurafenib-resistant clones (R6, B4 and D9). Changes were confirmed in n=3 independent replicates.

**FIGURE S4**

**
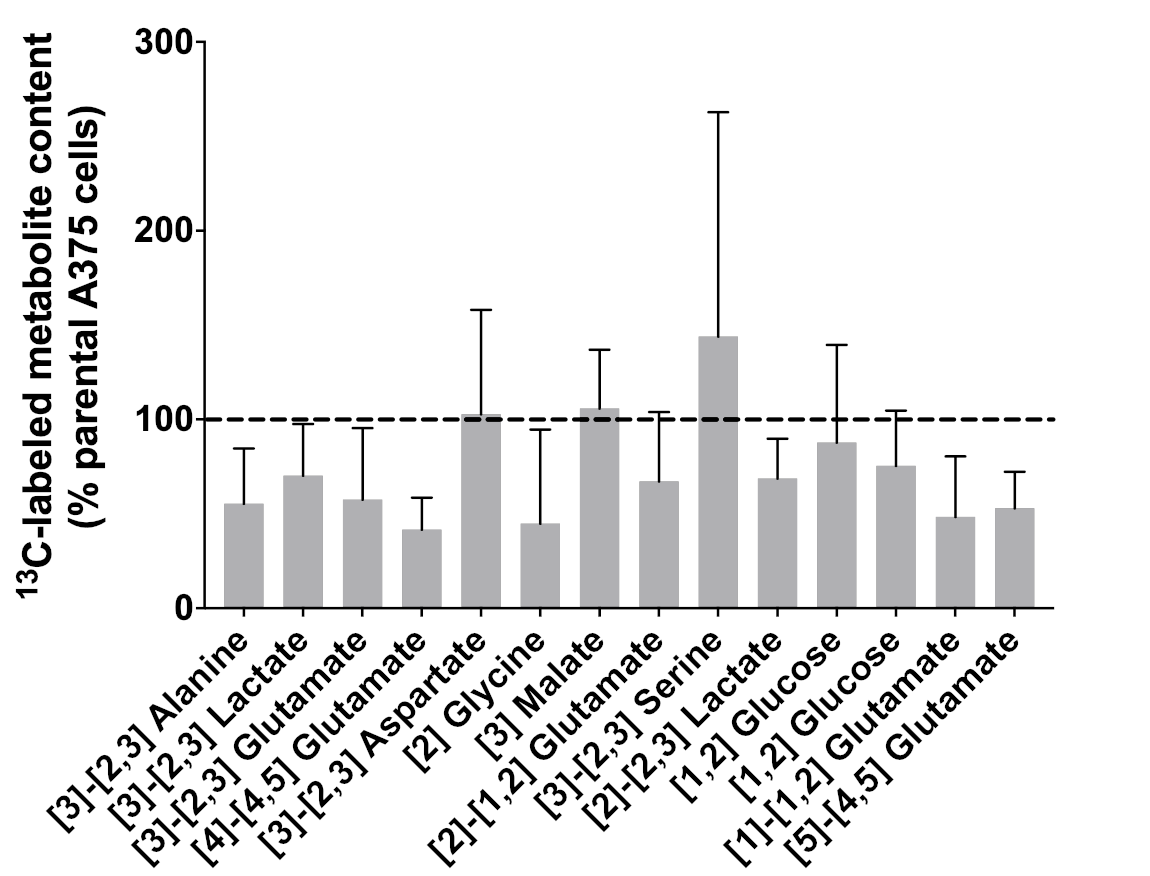
**

**Figure A: ^13^C NMR analysis in R6 cells with respect to the parental clone.** This graph is the equivalent to the one in Figure 4B of the main manuscript but showing the mean ± SD instead of the SE in order to better represent the data distribution.

**FIGURE S5**

**
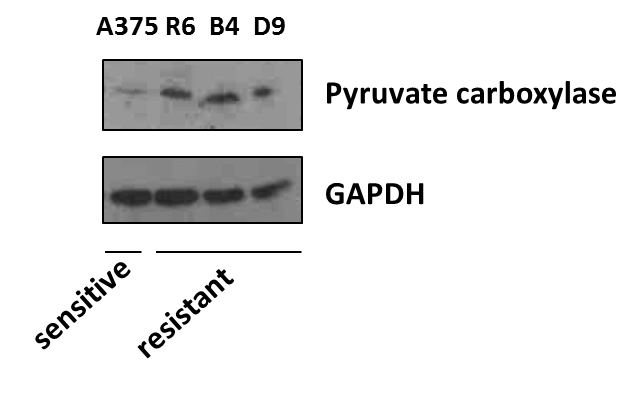
**

**Figure S5: Pyruvate carboxylase expression in acquired resistance to vemurafenib.** Protein expression of pyruvate carboxylase in A375 (A) cells and three vemurafenib-resistant clones (R6, B4 and D9). Changes were confirmed in n=3 independent replicates.

**SUPPLEMENTARY TABLES**

**TABLE S1:** ^1^H and ^31^P NMR main metabolites analysed in the aqueous phase of sensitive and resistant cell extracts (au/ cell number). For comparison of metabolites, Student t-test with Sidak-Bonferroni correction for multiple comparisons (P≤0.05) was applied. Data represent the mean ± SE. * Significant results (P≤0.05) are marked in bold.

| **^1^H NMR** | | | | |
| --- | --- | --- | --- | --- |
| **Cell metabolites (ppm)** | **Sensitive** | | **Resistant** | **P*** |
| BCCA (0.88 ppm) | 5.90 ± 0.98 | | 3.08 ± 0.57 | 0.228 |
| Lactate (1.33 ppm) | 14.72 ± 3.85 | | 7.84 ± 1.79 | 0.675 |
| Acetate (1.90 ppm) | 0.65 ± 0.11 | | 0.24 ± 0.03 | **0.007** |
| Glutamate (2.32 ppm) | 5.06 ± 0.39 | | 2.62 ± 0.26 | **0.002** |
| Glutamine (2.45 ppm) | 2.81 ± 0.32 | | 1.27 ± 0.19 | **0.008** |
| Glutathione (2.48 ppm) | 1.76 ± 0.17 | | 0.83 ± 0.12 | **0.007** |
| Aspartate (2.76 ppm) | 0.29 ± 0.17 | | 0.05 ± 0.01 | 0.501 |
| Creatine (3.03 ppm) | 2.11 ± 0.24 | | 1.64 ± 0.25 | 0.981 |
| Choline (3.21 ppm) | 0.24 ± 0.03 | | 0.19 ± 0.02 | 0.935 |
| Phosphocholine (3.22 ppm) | 8.53 ± 0.52 | | 5.54 ± 0.58 | 0.075 |
| GPCho (3.24 ppm) | 1.26 ± 0.10 | | 0.86 ± 0.09 | 0.241 |
| UDP-GlcNAC (5.49 ppm) | 0.09 ± 0.01 | | 0.04 ± 0.01 | **0.008** |
| Ribose (6.15 ppm) | 0.54 ± 0.05 | | 0.10 ± 0.04 | **0.053** |
| Fumarate (6.49 ppm) | 0.04 ± 0.004 | | 0.02 ± 0.003 | **0.001** |
|  |  | |  |  |
| **^31^P NMR** | | | | |
| **Cell metabolites (ppm)** | | **Sensitive** | **Resistant** | **P*** |
|  | |  |  |  |
| Phosphocholine (3.39 ppm) | | 6.82 ± 0.47 | 2.08 ± 0.61 | **0.0018** |
| GPE (0.38 ppm) | | 0.28 ± 0.02 | 0.07 ± 0.02 | **0.0003** |
| GPCho (-0.14 ppm) | | 0.79 ± 0.07 | 0.19 ± 0.04 | **0.00002** |
| Phosphocreatine (-2.99 ppm) | | 0.88 ± 0.09 | 0.56 ± 0.13 | 0.6577 |
| γ-NTP (-5.30 ppm) | | 5.65 ± 0.29 | 1.53 ± 0.41 | **0.0001** |
| α-NTP (-10.83 ppm) | | 6.04 ± 0.27 | 1.71 ± 0.46 | **0.0002** |
| β-NTP (-21.01 ppm) | | 6.22 ± 0.41 | 1.64 ± 0.45 | **0.0002** |

**TABLE S2:** ^1^H NMR and ^31^P NMR main metabolites analysed in the aqueous phase of resistant cell extracts (au/ cell number). For comparison of metabolites, Student t-test with Sidak-Bonferroni correction for multiple comparisons (P≤0.05) was applied. Data represent the mean ± SE. * Significant results (P≤0.05) are marked in bold.

| **^1^H NMR** | | | | |
| --- | --- | --- | --- | --- |
| **Cell metabolites (ppm)** | **R6** | | **B4 + D9** | **P*** |
| BCCA (0.88 ppm) | 2.09 ± 0.19 | | 3.51 ± 0.77 | 0.996 |
| Lactate (1.33 ppm) | 5.28 ± 2.50 | | 8.94 ± 2.30 | 0.999 |
| Acetate (1.90 ppm) | 0.17 ± 0.06 | | 0.27 ± 0.04 | 0.989 |
| Glutamate (2.32 ppm) | 2.16 ± 0.37 | | 2.82 ± 0.33 | 0.996 |
| Glutamine (2.45 ppm) | 1.14 ± 0.20 | | 1.33 ± 0.26 | 0.999 |
| Glutathione (2.48 ppm) | 0.88 ± 0.12 | | 0.80 ± 0.16 | 0.999 |
| Aspartate (2.76 ppm) | 0.04 ± 0.02 | | 0.05 ± 0.01 | >0.999 |
| Creatine (3.03 ppm) | 1.09 ± 0.18 | | 1.88 ± 0.31 | 0.948 |
| Choline (3.21 ppm) | 0.17 ± 0.05 | | 0.20 ± 0.02 | 0.999 |
| Phosphocholine (3.22 ppm) | 5.56 ± 0.51 | | 5.53 ± 0.83 | >0.999 |
| GPCho (3.24 ppm) | 0.57 ± 0.11 | | 0.98 ± 0.09 | 0.442 |
| UDP-GlcNAC (5.49 ppm) | 0.02 ± 0.01 | | 0.04 ± 0.01 | 0.992 |
| Ribose (6.15 ppm) | 0.27 ± 0.07 | | 0.34 ± 0.05 | 0.999 |
| Fumarate (6.49 ppm) | 0.01 ± 0.01 | | 0.02 ± 0.003 | >0.999 |
|  |  | |  |  |
| **^31^P NMR** | | | | |
| **Cell metabolites (ppm)** | | **R6** | **B4 + D9** | **P*** |
|  | |  |  |  |
| Phosphocholine (3.39 ppm) | | 4.66 ± 0.81 | 0.98 ± 0.20 | **0.002** |
| GPE (0.38 ppm) | | 0.15 ± 0.05 | 0.04 ± 0.01 | **0.028** |
| GPCho (-0.14 ppm) | | 0.34 ± 0.07 | 0.13 ± 0.02 | **0.028** |
| Phosphocreatine (-2.99 ppm) | | 1.02 ± 0.22 | 0.37 ± 0.09 | **0.028** |
| γ-NTP (-5.30 ppm) | | 3.09 ± 0.77 | 0.86 ± 0.14 | **0.016** |
| α-NTP (-10.83 ppm) | | 3.45 ± 0.92 | 0.96 ± 0.17 | **0.021** |
| β-NTP (-21.01 ppm) | | 3.28 ± 0.97 | 0.94 ± 0.16 | **0.028** |
